# A pan-cancer analysis of ABI3BP: a potential biomarker for prognosis and immunoinfiltration

**DOI:** 10.3389/fonc.2023.1159725

**Published:** 2023-05-01

**Authors:** Yan Feng, Fengying Tao, Han Qiao, Huaping Tang

**Affiliations:** ^1^ Department of Respiratory Medicine, Qingdao University, Qingdao, China; ^2^ Department of Oncology Qingdao Hospital, University of Health and Rehabilitation Sciences (Qingdao Municipal Hospital), Qingdao, China; ^3^ Department of Respiratory Medicine, Qingdao Hospital, University of Health and Rehabilitation Sciences (Qingdao Municipal Hospital), Qingdao, China

**Keywords:** ABI3BP, pan-cancer, prognosis, immune infiltration, tumor target

## Abstract

**Objective:**

ABI Family Member 3 Binding Protein (ABI3BP) is an extracellular matrix protein that affects the carcinogenesis of lung and esophageal cancer. However, the relevance of ABI3BP in different forms of cancer is uncertain.

**Methods:**

ABI3BP expression was interpreted using the Cancer Genome Atlas (TCGA) database, the Genotype Tissue Expression Atlas (GTEx) database, the Human Protein Atlas (HPA) database, the Cancer Cell Line Encyclopedia (CCLE) database, and immunohistochemistry. The R programming language was used to analyze the association between ABI3BP expression and patient prognosis and evaluate the relationship between ABI3BP and the immune characteristics of tumors. Using the GDSC and CTRP databases, a drug sensitivity analysis of ABI3BP was conducted.

**Results:**

ABI3BP mRNA expression was shown by differential analysis to be down-regulated in 16 tumor types relative to normal tissues, corresponding with its protein expression level as determined by immunohistochemistry. Abnormal expression of ABI3BP accurately predicts the prognosis of patients with renal chromophobe carcinoma (KICH), mesothelioma (MESO), and pancreatic adenocarcinoma (PAAD). Meanwhile, aberrant expression of ABI3BP was associated with immune checkpoints, TMB, MSI, tumor purity, HRD, LOH, and drug sensitivity. A correlation between ABI3BP expression and the amount of infiltration of several immune-related cells in pan-cancer was determined by Immune Score, Stromal Score, and Estimated Score.

**Conclusion:**

Our results show that ABI3BP might be employed as a molecular biomarker to predict prognosis, treatment susceptibility, and immunological response in patients with pan-cancer.

## Introduction

Cancer is one of the main global causes of mortality (1). Therefore, it is crucial to find biomarkers and possible tumor prognostic regulators. Therapies and immunotherapies that target tumors have recently rendered novel patient-inclined directions, with the aim to increase patient survival by activating T cells and regulating T cell infiltration in patients (2, 3). In addition, research indicates that immunotherapy stimulates the body’s immune response to tumors (4–6). Therefore, a better understanding of the interaction and molecular mechanism between tumor cells and immune cells could yield new immunotherapy targets for tumor patients.

ABI3BP, an extracellular matrix protein regulated by Akt and ubiquitin (7), was found in a yeast two-hybrid screen utilizing the c-Abl binding protein ABI3, also known as TARSH and eratin, as bait (8). ABI3BP promotes cell adhesion and extracellular matrix assembly (7). It has been reported that elevated ABI3BP expression in esophageal cancer cells inhibits cell apoptosis and promotes cell proliferation (9). It has also been reported that ABI3BP participates in cellular senescence *via* the p53-dependent p21 (cyclin-dependent kinase inhibitor) pathway to inhibit tumor progression (10). However, there are no studies on the effect of ABI3BP on immune infiltration and prognosis in pan-cancer.

With the recent development of high-throughput sequencing technologies, bioinformatics has emerged due to the detection of a vast amount of microscopic information in cancerous and healthy tissues, and the expansion of numerous public databases. Bioinformatics primarily employs mathematical, statistical, and computational techniques to process and analyze biological data, such as genomics and proteomics. Bioinformatics is currently utilized extensively in the study of cancer and other diseases.

This study analyzed the differences in ABI3BP mRNA and protein expression levels in pan-cancer tissues using the TCGA, GTEx, CCLE, and HPA databases. We also explored its predictive value for tumor prognosis and its role in various tumor-related immune responses to give a theoretical framework for future study into possible immunotherapy targets for tumors.

## Methods

### Data collection and collation

Two databases, GTEx (https://gtexportal.org/) and TCGA (https://tcga-data.nci.nih.gov/tcga/), were used to gather the ABI3BP mRNA expression matrix and clinically relevant data for each tumor tissue and normal tissue. Using GTEx and TCGA data, we evaluated the changes in ABI3BP expression levels between normal and cancerous tissues. ABI3BP protein expression levels were collected from the HPA database (https://www.proteinatlas.org/). ABI3BP mRNA expression levels were obtained from the CCLE database (https://portals.broadinstitute.org/ccle). The p< 0.05 was used to determine expression differences between tumor and normal tissue samples. R software (version 4.0.2, https://www.Rproject.org) was used for data processing, and block plots were generated using the “ggplot2” R package.

### Human samples

The cancer and adjacent normal tissue excised from tumor patients were collected in Qingdao Municipal Hospital. This study was conducted in strict accordance with the principles expressed in the Declaration of Helsinki and have reviewed and approved by the Ethics Committee of the Qingdao Municipal Hospital(2022yxy071). The lesion tissue samples of the selected patients were collected. The samples were fixed, dehydrated, transparent, waxed, embedded, sectioned, and the sectioned samples were obtained. excluding samples with exfoliation and incomplete clinical data. remaining samples (a total of 8 pairs) were used for immunohistochemical result analysis.

### Immunohistochemistry

The ABI3BP expression-measuring antibody was purchased from Elabscience (ABI3BP: E-AB-10970). Tissue sections were cut from formalin-fixed paraffin-embedded blocks containing representative tumors (4-mm-thick). The prepared section samples were baked in a 60°C oven for 1h and then successively placed in xylene I for 10min and xylene II for 10min to ensure complete tissue dewaxed. Then they were removed and placed in absolute ethanol, 95% ethanol, and 75% ethanol successively for 2 minutes each, and finally washed with pure water. Citric acid antigen repair was then performed and the sections were incubated overnight with rabbit ABI3BP resistance (1:100) at 4°C. Normal goat serum was used as a negative control. These sections were eventually stained with hematoxylin, dried with gradient alcohol, rendered transparent with xylene, and then sealed with a neutral glue.

### Analysis of prognosis and diagnostic value

The researchers examined the link between ABI3BP expression and patient prognosis as measured by overall survival (OS), disease specific survival (DSS), disease free interval (DFI), and progression free interval (PFI). Survival analysis was performed using Kaplan-Meier curves and log-rank testing for each kind of cancer. To plot survival curves, the “survival” and “survminer” R packages were used. Furthermore, we investigated the relationship between ABI3BP expression and pan-cancer survival using the “forestplot” R program. The diagnostic accuracy of ABI3BP was evaluated by ROC curve analysis based on sensitivity and specificity using the “pROC” and “ggplot2” packages, respectively.

### Analysis of immune infiltration

The R package “immunedeconv” algorithms TIMER, CIBERSORT, QUANTISEQ, XCELL, MCPCOUNTER, and EPIC were used to estimate immune infiltration. Through the ESTIMATE algorithm, the level of immune cell infiltration (ImmuneScore) and the abundance of stromal components (StromalScore) in each TCGA sample were compared to an Estimated Score. Filtering the samples with zero expression, analyzing each expression value using the log2 (x + 0.001) transformation, and displaying the data as heat maps. Spearman correlation analysis was used to examine the relationship between ABI3BP, immunological checkpoints, and tumor heterogeneity; a p-value of less than 0.05 after adjustment was deemed statistically significant.

### Gene set analysis

Using GEPIA2 (http://gepia.cancer-pku.cn/), we identified the top 100 ABI3BP-related genes. In the “correlation analysis” module of GEPIA2, the Pearson correlation test was performed on the top 10 ABI3BP-related genes, and correlation coefficients and scatter plots were obtained. GSCA database (11) (http://bioinfo.life.hust.edu.cn/GSCA) to examine the GSVA enrichment score of the top 20 ABI3BP-related genes, the relationship between GSVA enrichment score and immune infiltration, and the effect of GSVA score on gene set prognosis. Simultaneously, we examined the analysis of CNV mutation on tumor immune infiltration and patient prognosis by analyzing CNV mutations.

### Drug sensitivity

A circular correlation analysis plot was displayed based on the Genomics of Drug Sensitivity in Cancer (GDSC) database (https://www.cancerrxgene.org/) and the Cancer Therapeutics Response Portal (CTRP) database (https://portals.broadinstitute.org/ctrp/) for drug susceptibility and drug resistance analyses. We have collected 481 small molecules of IC1001 and their corresponding mRNA gene expression in 50 cell lines from the CTRP. The correlation between ABI3BP expression and drug IC50 was obtained by combining mRNA expression data with drug susceptibility data and using Pearson correlation analysis. P values were adjusted by FDR. Similarly, we have collected IC860 of 265 small molecules and their corresponding mRNA gene expression from 50 cell lines in GDSC. The correlation between ABI3BP expression and drug IC50 was obtained by Pearson correlation analysis. Then filter correlation | cor | > 0.150 drugs, and draw circular diagram to show correlation between ABI3BP and drugs.

### Statistical analysis

The data is shown as the mean ± SEM. Using the Student’s t-test, the two unpaired groups were evaluated statistically.

## Results

### ABI3BP expression

**Figure 1:**
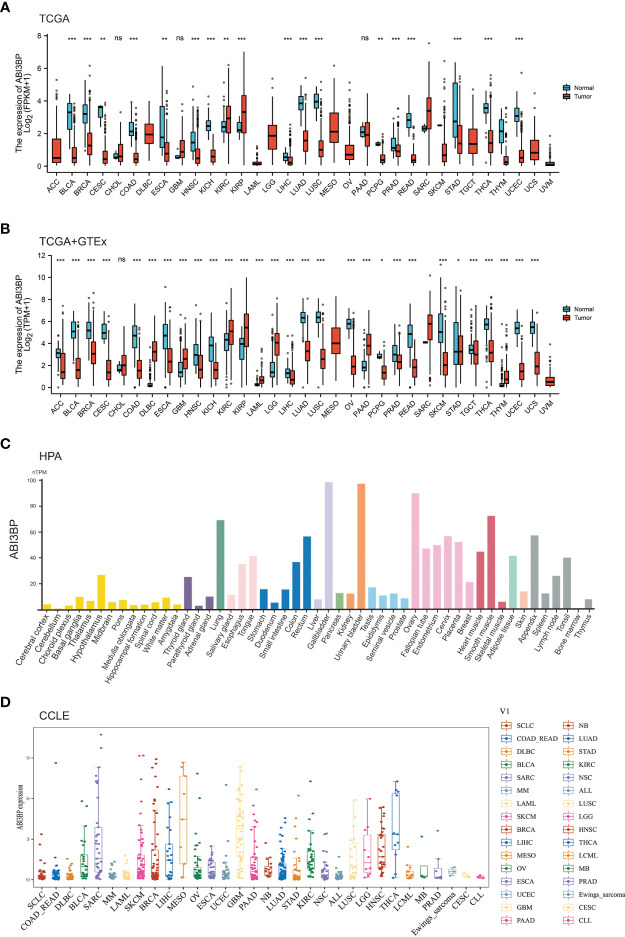
ABI3BP expression profile. **(A)** ABI3BP expression levels in cancer and adjacent normal tissues from TCGA database. **(B)** Differences in ABI3BP expression levels between tumor and normal tissues in TCGA and GTEx databases. **(C)** ABI3BP protein expression level in HPA database. **(D)** ABI3BP expression level of tumor cell lines in CCLE database. *p < 0.05, **p < 0.01, ***p < 0.001.

We started by analyzing the levels of ABI3BP expression in diverse malignancies. The examination of TCGA datasets indicated that ABI3BP expression in normal tissues was greater than in BLCA, BRCA, CESC, COAD, ESCA, HNSC, KICH, LIHC, LUAD, LUSC, PCPG, PRAD, READ, STAD, THCA, and UCEC tissues; only KIRC and KIRP tissues were higher than in normal tissues (**Figure 1A**). Since the TCGA database contains fewer normal tissue data, we combined the normal tissue data from the GTEx database with the TCGA database’s tumor data. The results indicated that the expression level of ABI3BP in tumor tissues was significantly lower than that of normal tissues, and it was higher in a small percentage of tumors. For example, in ACC, BLCA, BRCA, CESC, COAD, ESCA, HNSC, KICH, LIHC, LUAD, LUSC, OV, PCPG, PRAD, READ, SKCM, STAD, TGCT, THCA, UCEC and UCS, ABI3BP expression is significantly lower than in normal tissue (**Figure 1B**). Using the HPA database, we also analyzed the protein levels of ABI3BP. ABI3BP expression levels were higher in the gallbladder, urinary bladder, ovary, smooth muscle, and lungs, but lower in bone marrow, cerebellum, and parathyroid glands (**Figure 1C**). The CCLE database displays ABI3BP expression levels in 32 tumor cell lines, with the MESO cell line expressing the highest levels and the CLL cell line displaying the lowest levels (**Figure 1D**).

**Figure 2:**
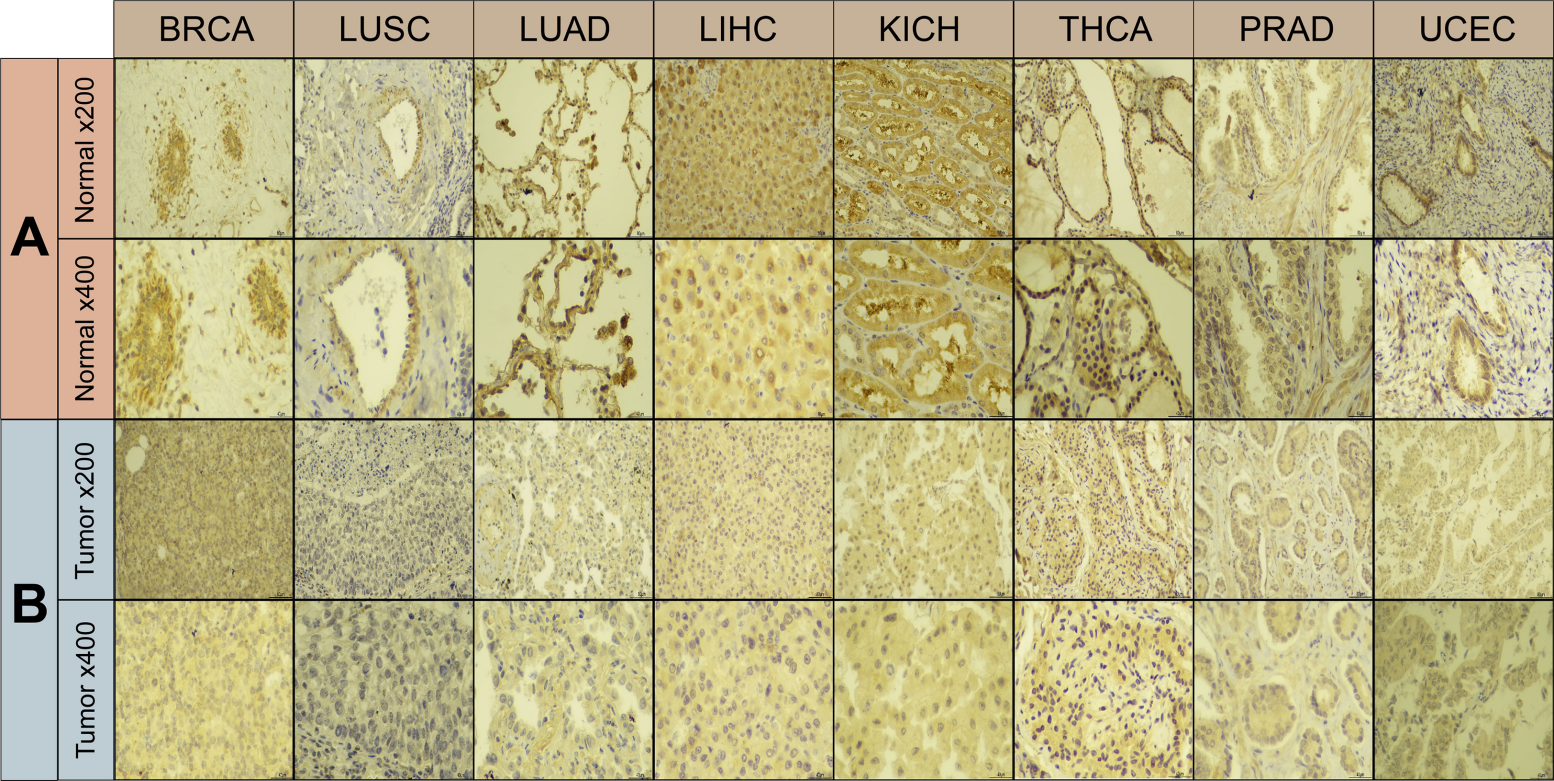
Immunohistochemical results of ABI3BP. **(A, B)** ABI3BP protein expression levels in BRCA, LUAD, LUSC, LIHC, KICH, THCA, PRAD, UCEC and corresponding normal tissues. Original magnification, ×200 and ×400.

Using immunohistochemistry labeling, the protein expression levels in different cancers and associated normal tissues were evaluated. ABI3BP protein expression was diminished in a variety of solid tumors, including BRCA, LUSC, LUAD, LIHC, KICH, THCA, PRAD, and UCEC, as indicated by the protein levels of ABI3BP in various cancers (**Figures 2A, B**). These results indicate that ABI3BP expression is suppressed in a variety of cancer types, suggesting that ABI3BP may play a crucial role in cancer detection.

### Diagnostic and prognostic analyses of ABI3BP

**Figure 3:**
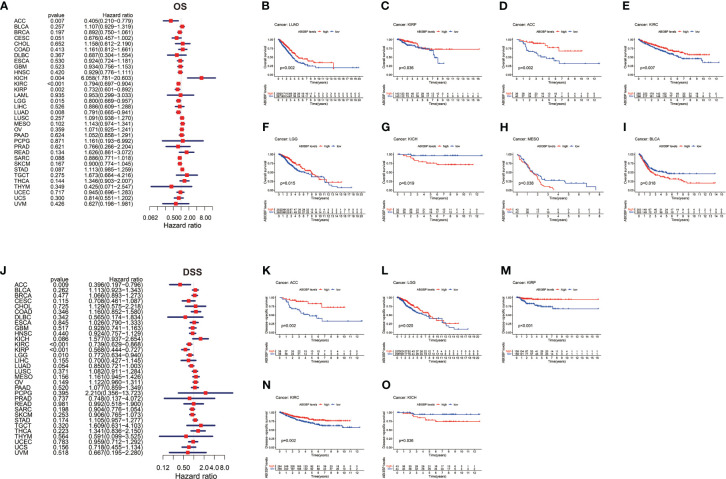
Univariate Cox regression analysis of ABI3BP. **(A)** Forest plot shows the results of univariate cox regression of ABI3BP on OS in TCGA database. **(B-I)** The K-M survival curve showed the OS prognosis of LUAD, KIRP, ACC, KIRC, LGG, KICH, MESO and BLCA patients in the ABI3BP high and low expression groups. **(J)** Forest plot shows the univariate cox regression results of ABI3BP on DSS in TCGA database. **(K-O)** The K-M survival curve showed the DSS prognosis survival of ACC, LGG, KIRP, KIRC and KICH patients in the ABI3BP high and low expression groups.

**Figure 4:**
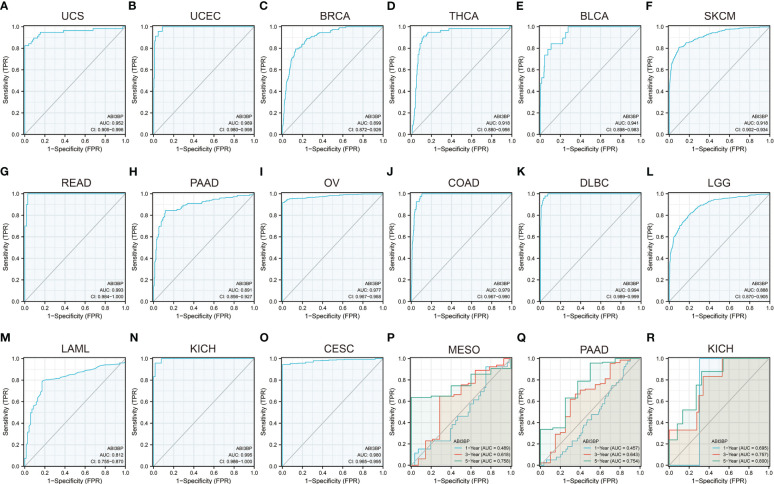
Diagnostic and prognostic value of ABI3BP in patients. **(A-O)** ROC curve of ABI3BP for the prognosis of UCS, UCEC, BRCA, THCA, BLCA, SKCM, READ, PAAD, OV, COAD, DLBC, LGG, LAML, KICH, CESC patients. **(P-R)** Time-dependent ROC analysis of ABI3BP for 1 -, 3 -, and 5-year survival rates in MESO, PAAD, and KICH patients.

Using Cox proportional hazard models and Kaplan Meier analyses, the diagnostic potential of ABI3BP was evaluated. The forest plot shows the univariate cox regression results of ABI3BP to OS in TCGA pan-carcinoma (**Figure 3A**). ABI3BP posed a low risk for ACC, KIRC, KIRP, LGG, and LUAD but a high risk for KICH (HR =6.058) (**Figure 3A**). High ABI3BP expression was related with a favorable outcome in patients with LUAD, ACC, KIRP, KIRC, and LGG, as shown by Kaplan Meier curves (**Figures 3B-F**). Its high expression was associated with an unfavorable outcome in KICH, MESO, and BLCA patients (**Figures 3G-I**). **Figure 3J** displays ABI3BP’s univariate cox regression results for DSS. ABI3BP is a low-risk factor for ACC, KIRC, KIRP, and LGG. From the K-M curve, a high expression of ABI3BP was related with a better prognosis in ACC, KIRP, KIRC, and LGG patients, but also with poor prognosis in KICH patients (**Figures 3K-O**). **Supplementary Figure 1A** depicts the univariate cox regression results of ABI3BP for DFI; ABI3BP is a low-risk factor for KIRP, LIHC, and LUAD. High ABI3BP expression was related with a better prognosis in LIHC patients, but a bad prognosis in ESCA patients (**Supplementary Figures 1B, C**). Lastly, the Cox regression analysis of PFI revealed that ABI3BP was a risk factor with a lower association with ACC, KIRP, LGG, LIHC, and LUAD. The K-M curve demonstrated that ACC, KIRP, KIRC, and LIHC patients with high ABI3BP expression had a favorable prognosis (**Supplementary Figures 1E-H**). High ABI3BP expression was associated with a poor prognosis for SARC and KICH patients (**Supplementary Figures 1I, J**). In order to examine further the diagnostic and prognostic capacity of ABI3BP on tumor patient prognosis, we performed ROC analysis on the TCGA database. Notably, the AUC of READ, DLBC, and KICH patients is greater than 0.99 *(* **Figures 4G, K, N**). We further observed that ABI3BP had a good prediction ability for UCS, UCEC, BRCA, THCA, BLCA, SKCM, READ, PAAD, OV, COAD, DLBC, LGG, LAML, and CESC prognoses; moreover, the AUCs were all above 0.80 (**Figures 4A-O**). Then, we reviewed data from the TCGA and GTEx databases, and a time-dependent ROC analysis indicated that ABI3BP had superior predictive power for 5-year survival in patients with KICH (AUC = 0.800) (**Figure 4R**), MESO (AUC = 0.758) (**Figure 4P**), and PAAD patients (AUC = 0.754) (**Figure 4Q**). Our findings indicate that abnormal ABI3BP expression may serve as a cancer biomarker.

### Relationship between ABI3BP and clinical data

**Figure 5:**
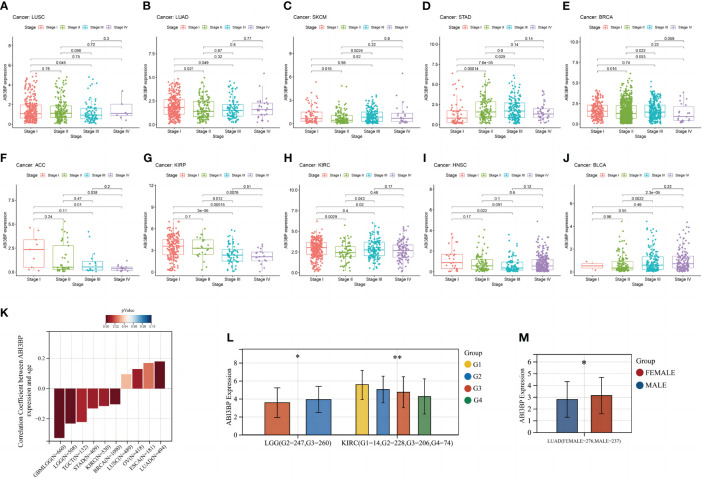
Relationship between ABI3BP and clinical case data in pan-cancer. **(A-J)** ABI3BP expression in LUSC, LUAD, SKCM, STAD, BRCA, ACC, KIRP, KIRC, HNSC, BLCA cancer stages. **(K)** Relationship between ABI3BP expression and age in different cancers. **(L)** ABI3BP expression in different G stages of cancer. **(M)** Relationship between ABI3BP expression and sex in LUAD. *, p < 0.05; **, p < 0.01.

We also examined ABI3BP expression at various cancer stages. In 10 malignancies, we found a strong correlation between ABI3BP expression levels and tumor stages. We also found that ABI3BP expression was lower in the later stages of the majority of tumors, especially stages III and IV, including LUSC, LUAD, BRCA, ACC, KIRP, and HNSC (**Figures 5A, B, E, F, G, I**). However, the expression of ABI3BP was higher in the later stages of SKCM, STAD, KIRC, and BLCA (**Figures 5C, D, H, J**). Age was also identified as a key factor impacting ABI3BP expression. ABI3BP expression was negatively correlated with age in most tumor tumors, including GBMLGG, LGG, TGCT, STAD, KIRC, and BRCA (**Figure 5K**). In contrast, ABI3BP expression was positively correlated with age in LUSC, OV, ESCA, and LUAD (**Figure 5K**). Likewise, we determined notable differences in ABI3BP expressions against their Grade amongst eight tumors; ABI3BP expression levels were considerably lower in LGG and KIRC tumors with higher Grades (**Figure 5L**). Likewise, we discovered that female patients with LUAD and BRCA had higher ABI3BP expression than male patients (**Figure 5M**). The data suggest that ABI3BP may be associated with the development and progression of malignancies.

### Relationship between ABI3BP and immune cell infiltration

**Figure 6:**
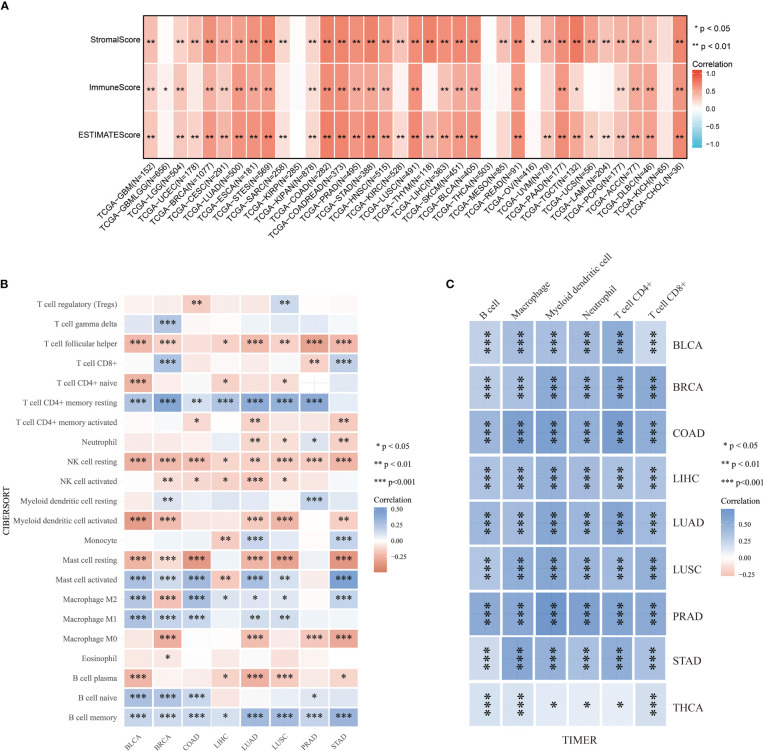
Analysis of tumor immune infiltration score for ABI3BP. **(A)** ABI3BP tumor estimation score analysis, stromal score analysis and tumor immune infiltration analysis. **(B, C)** CIBERSORT and TIMER algorithms analyzed the level of immune cell infiltration. *, p < 0.05; **, p < 0.01; ***, p < 0.001.

The tumor immune microenvironment (TIME) is significantly influenced by tumor-infiltrating components and is one of the most important predictors of tumor immunotherapy efficacy. The Estimate algorithm is used as a tool to predict tumor purity and invasive stroma/immune cells in tumor tissue using gene expression data. We used it to analyze the relationship between ABI3BP expression and immunoinfiltration and produced three scores:1) Stroma score (which captures the presence of stroma in tumor tissue); 2) Immune score (representing the infiltration of immune cells in tumor tissue); 3) Estimate score (infer tumor purity). We first used Stroma score, Immune score, and Estimate score to investigate the association between ABI3BP expression and immune infiltration (**Figure 6A**). The top three tumors with significant correlation between ABI3BP and Stroma score were TGCT, THYM, and COAD. And ABI3BP was associated with a significant Immune score the top three for COAD, COADREAD and LUSC tumor. The top three tumors with significant correlations between ABI3BP and Estimate score were COAD, COADREAD, and STES (**Figure 6A**). In addition, we performed a correlation analysis using immune cell infiltration data from six distinct sources, including CIBERSORT (**Figure 6B**), TIMER (**Figure 6C**), MCPCOUNTER (**Supplementary Figure 2A**), xCell (**Supplementary Figure 2B**), EPIC (**Supplementary Figure 2C**), and QuanTIseq (**Supplementary Figure 2D**). Based on the obvious relationship between ABI3BP and immune response, we performed a pan-cancer analysis of the relationship between ABI3BP expression and the level of immune infiltration according to the TIMER database. As shown in **Figure 6B**, the ABI3BP expression was significantly correlated with the abundance of infiltrating immune cells: B cells in seven cancers, NK cells in seven cancers, Mast cells in six cancers, Macrophage M2 cells in six cancers, and T cell CD4+ memory resting in six cancers. The TIMER tool was further used to examine the relationship between ABI3BP expression and infiltration of different types of immune cell subtypes. Among the six immune cell subtypes, a significant positive correlation was found for the ABI3BP expression with these subtypes of BLCA, BRCA, COAD, LIHC, LUAD, LUSC, PRAD, STAD, and THCA (**Figure 6C**). The results of the MCPCOUNTER and EPIC algorithms were similar: Myeloid dendritic cells and Endothelial cells were associated with the strongest positive correlation between ABI3BP expression in various cancers was found (**Supplementary Figures 2A, C**). The xCell algorithm also provided the corresponding analysis: the strongest negative correlation between T cell CD4+ Th1, Th2 and T cell CD4+ central memory cells and the presence of ABI3BP expression in various cancers (**Supplementary Figure 2B**).

### Correlation of ABI3BP expression with immune-related biomarkers

**Figure 7:**
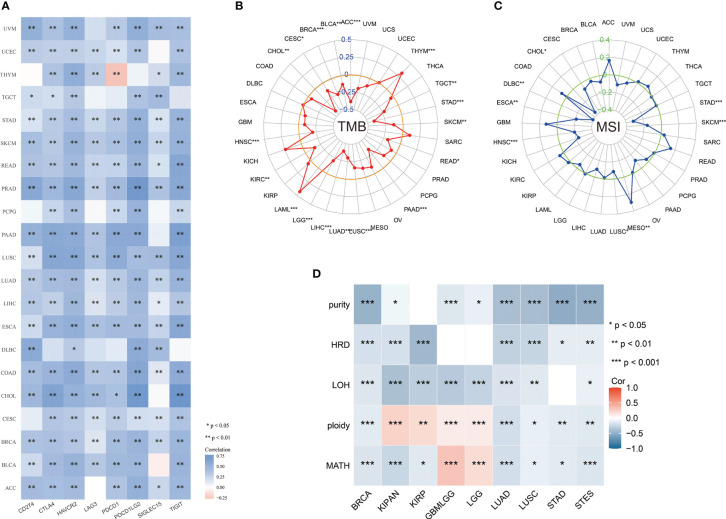
Relationship between ABI3BP expression and immune checkpoints and tumor heterogeneity. **(A)** Relationship between ABI3BP expression and immune checkpoints. Radar plot of ABI3BP expression in relation to TMB **(B)** and MSI **(C)**. **(D)** Relationship between ABI3BP expression and genomic heterogeneity. *, p < 0.05; **, p < 0.01; ***, p < 0.001.

Immune checkpoints are a group of molecules with the capacity to regulate T cells, which directly inhibit and stimulate immune cell functions, contribute to tumor cell immune evasion, and result in tumor metastasis and invasion (12). CD274, also known as PD-L1, is expressed by tumor cells in the tumor microenvironment. Binding of CD274 to PD-1 leads to tyrosine phosphorylation of the intracellular structural domain of PD-1 and recruitment of the tyrosine phosphatase SHP-2, which reduces activation signals downstream of the TCR pathway as well as T-cell activation and cytokine production (13). TIGIT is a type I transmembrane protein, and *in vitro* blockade of TIGIT enhances the activation and degranulation of NK and T cells, and also increases the secretion of cytokines such as IFN-γ (14). LAG-3 (lymphocyte activation gene 3) and CD4 are homologous proteins that are expressed in a variety of immune cells and negatively regulates T cell function (15). Immune checkpoint analysis revealed that ABI3BP expression in most tumors was significantly correlated with CD274, CTLA4, HAVCR2, LAG3, PDCD1, PDCD1LG2, TIGIT, and SIGLEC15 (**Figure 7A**). It shows that ABI3BP may be an immunotherapy target. To perform a comprehensive and trustworthy immunocorrelation analysis, we performed TMB, MSI, MMR, NEO, purity, and HRD score analysis for ABI3BP. TMB, defined as the number of mutations in cancer cells’ DNA, can improve the predictive accuracy of immunotherapy outcomes and aid in selecting the most effective immune checkpoint inhibitors (16). The expression of ABI3BP in patients with ACC, BLCA, BRCA, CHOL, CESC, TGCT, STAD, SKCM, READ, PAAD, LUAD, LUSC, LGG, LIHC, KIRC, and HNSC is significantly negatively correlated with TMB (**Figure 7B**); the opposite goes with THYM and LAML patients. Microsatellite instability (MSI) status has been evaluated as a potential predictive biomarker in cancer immunotherapy. Numerous studies have demonstrated that MSI tumor patients exhibit significant immunotherapy, prognosis, and chemosensitivity heterogeneity (17). ABI3BP expression in CHOL, DLBC, ESCA, HNSC, LUSC, SKCM, STAD, and MESO correlates significantly with MSI (**Figure 7C**). It is generally recognized that patient-collected tumor tissue includes both tumor cells and non-tumor cells, which may dilute the purity of tumor cells and impact cancer histological subtype identification. Therefore, it is necessary to assess tumor purity. We found a significant correlation between ABI3BP and tumor purity in BRCA, STAD, and STES after a comprehensive examination of the relationship between abnormal gene expression of ABI3BP and tumor purity (**Figure 7D**). LOH, HRD, ploidy, and MATH facilitate the study of the evolution of the cancer genome and intratumoral heterogeneity, which is the basis for drug resistance to numerous cancer treatments (18). LOH (loss of heterozygosity) can cause the loss of the entire gene and its nearby chromosomal regions (19). HRD (Homologous recombination deficiency) is a key indicator of various cancer treatment options and prognosis, resulting in specific, quantifiable, and stable genome changes (20). Ploidy is a marker of cancer and is closely related to chromosome instability in cancer development. Estimating the purity and ploidy of tumors is beneficial to the evolution of cancer genomes and the study of intra-tumor heterogeneity (21). The MATH (Mutant-allele tumor heterogeneity) could effectively deviate from the distribution of MAF values representing tumor-specific mutation sites. The larger the MATH value, the higher the tumor heterogeneity (22). Notably, ABI3BP negatively correlates with LOH, HRD, ploidy, and MATH in BRCA, LUAD, LUSC, and STES (**Figure 7D**). The aforementioned results demonstrate a correlation between ABI3BP expression and immune cell infiltration.

### Correlation between ABI3BP expression and immune-related genes

**Figure 8:**
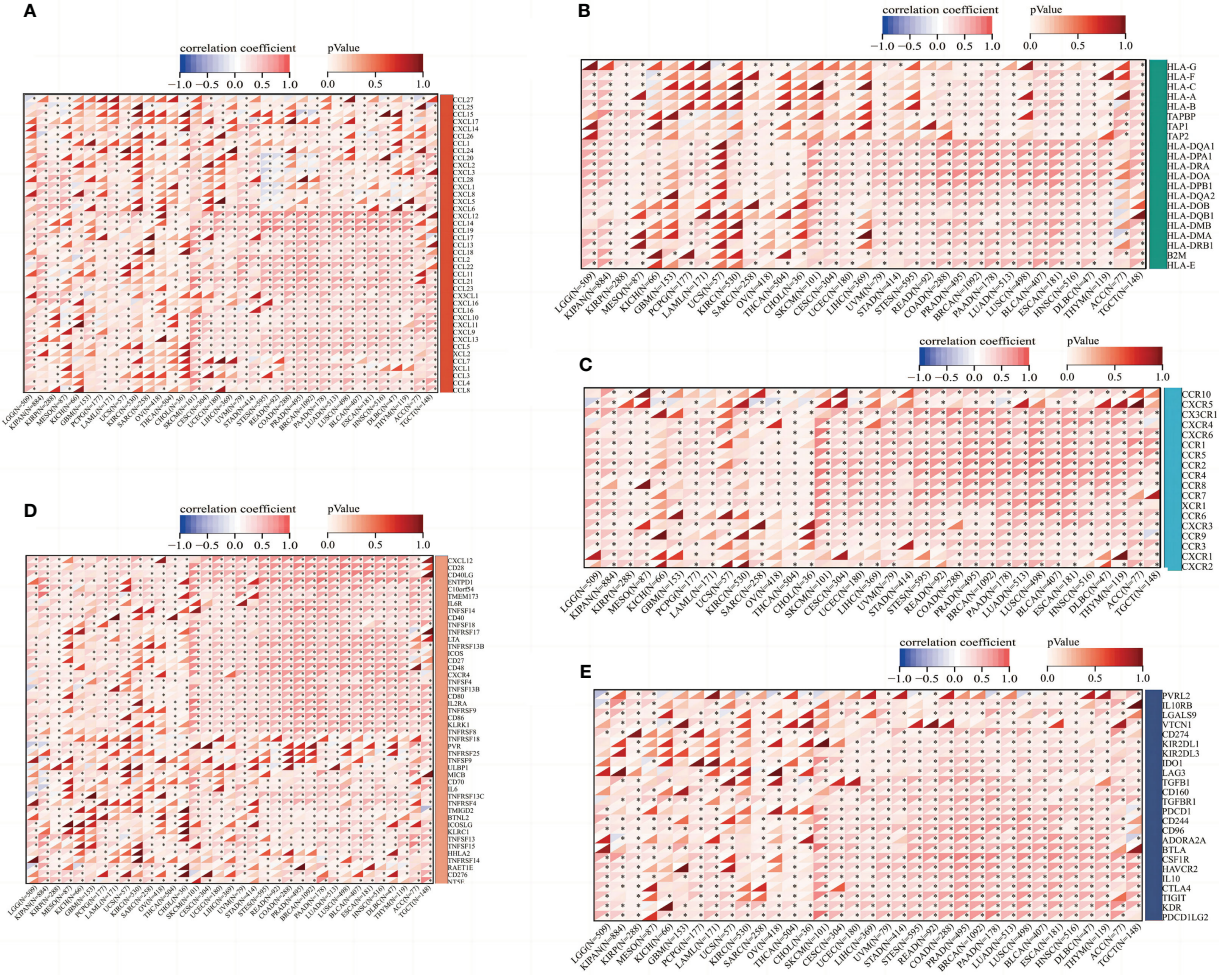
Correlation between ABI3BP expression and immune-related genes. **(A)** Correlation between the expression of ABI3BP and chemokine genes. **(B)** Correlation between ABI3BP expression and MHC genes. **(C)** Correlation between ABI3BP expression and receptor genes. **(D, E)** Correlation of ABI3BP expression with immunosuppressive agents and immunostimulants. *, p < 0.05.

Afterward, we determined possible correlations on immune-associated pan-cancer genes by ABI3BP expressions. According to genetic correlation analysis, in almost all types of cancer, the expression of ABI3BP is correlated with most chemokines such as CXCL9 and CXCL12 (**Figure 8A**), MHC genes such as HLA-DRA and HLA-DOA (**Figure 8B**), receptors such as CCR4 and CCR2(**Figure 8C**), and immune stimulants such as CD28 and CD40(**Figure 8D**) and immunosuppressive agents such as PDCD1LG1 and KDR (**Figure 8E**). In addition, most of the genes analyzed were positively correlated with ABI3BP expression. These results indicate that ABI3BP expression is significantly correlated with immune-related genes.

### ABI3BP-related gene analysis

**Figure 9:**
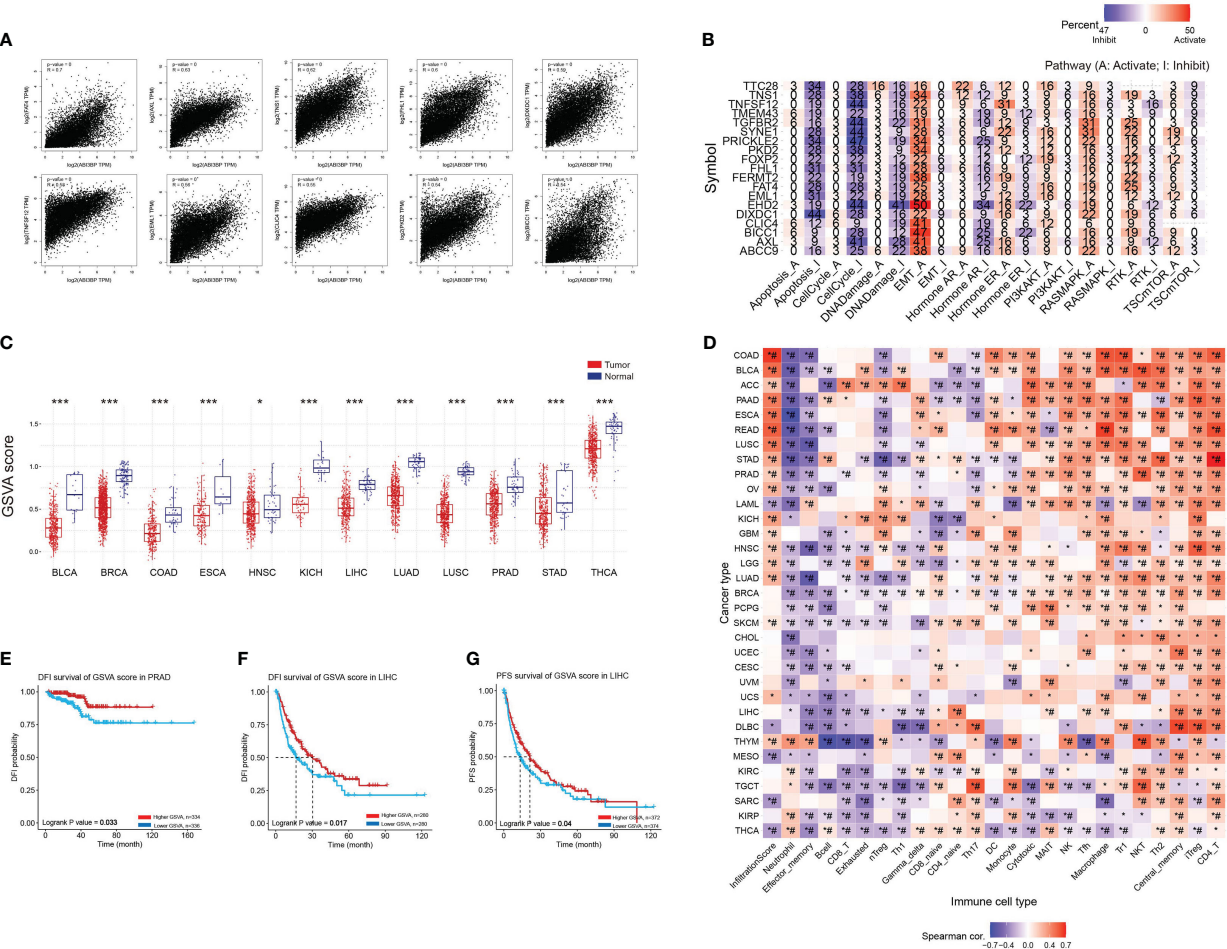
GSVA scores of ABI3BP-related genes and their association with immune infiltration. **(A)** The top 20 genes with the strongest interaction with ABI3BP were screened in the GEPIA website, and the scatter plots of the associations of the top 10 genes are shown. **(B)** roles of top 20 genes in cancerrelated pathways. **(C)** GSVA scores of the top 20 genes. **(D)** relationship between gene sets and immune infiltrating cells. **(E–G)** Relationship between GSVA score of gene sets and patient outcomes. *, p < 0.05; ***, p < 0.001; #, FDR ≤ 0.05.

We used the GEPIA website to identify the top 100 genes associated with ABI3BP, and a scatter plot of the ten most correlated genes is displayed (**Figure 9A**). Using the top 20 genes as the study gene set, it was discovered that they were significantly linked to the inhibition of biological processes such as apoptosis, cell cycle, and DNA damage, as well as the activation of tumor EMT (**Figure 9B**). Next, we performed GSVA enrichment scores on gene sets and discovered that gene sets were less expressed in multiple tumors than in adjacent normal tissues (**Figure 9C**). Moreover, except for a few tumors, Tfh, Macrophage, Th1, NKT, Th2, Central_memory, iTreg, and CD4_T were significantly positively correlated with gene set GSVA (**Figure 9D**). In addition, in PRAD tumors, the DFI of the patient and the DFI and PFS of LIHC patients are enhanced proportionally to the GSVA score of the gene set (**Figures 9E-G**). This shows that genes linked to ABI3BP are engaged in cancer-associated functional states and govern the immune responses of tumor cells, influencing the prognosis of patients.

**Figure 10:**
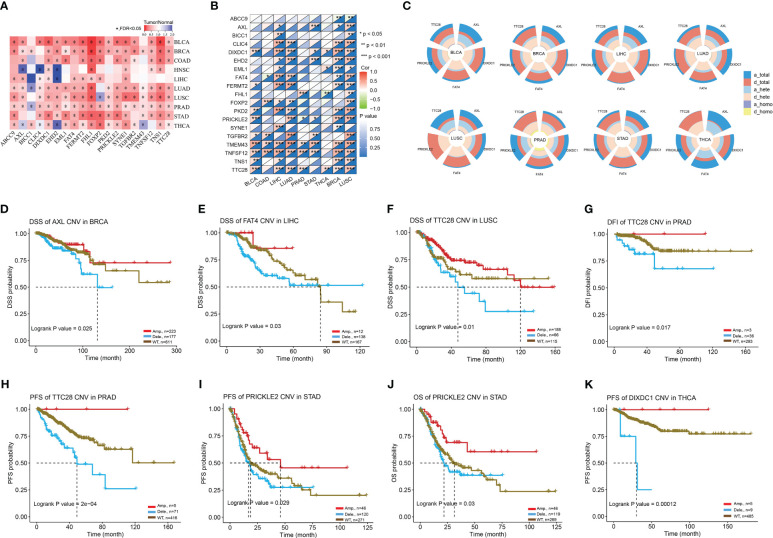
CNV mutation landscape of ABI3BP-related genes. **(A)** ABI3BP-related gene expression levels in pan-cancer patients. **(B)** Correlation between CNV mutations and mRNA levels of ABI3BP-related genes. **(C)** Percentage of CNV in AXL, DIXDC1, FAT4, PRICKLE2, and TTC28. **(D-K)** Association between CNV mutations and prognosis in patients with BRCA, LIHC, LUSC, PRAD, STAD, and THCA. *, p < 0.05; **, p < 0.01; ***, p < 0.001.

Next, we investigated the influence of gene sets on the immune response in tumors. CNV or copy number variation can have major biological consequences for species-specific genome composition, species evolution and phylogeny, and gene expression and regulation in certain genomic areas (23). An aberrant CNV is a crucial biological process in the development of several illnesses, including cancer. When we downloaded the mRNA expression data and CNV raw data from the TCGA database (**Figure 10A**), we noticed that the majority of genes in the gene set were expressed less in tumors than in normal tissues, and that their mRNA expression levels were substantially linked with CNV mutations (**Figure 10B**). Further investigation revealed that the CNV mutation type of AXL was primarily copy number amplification (**Figure 10C**), associated with a good prognosis in BRCA patients (**Figure 10D**). Meanwhile, FAT4, TTC28, PRICKLE2, and DIXDC1 were primarily copy number deletions (**Figure 10C**), associated with poor prognosis of LIHC (**Figure 10E**), LUSC (**Figure 10F**), PRAD (**Figures 10G, H**), STAD (**Figures 10I, J**), and THCA (**Figure 10K**). In conclusion, ABI3BP-associated CNV mutations are crucial for tumorigenesis. The instability of the tumor genome enables cancer cells to generate genetic alterations that drive tumor progression, and mutations in ABI3BP-related gene sets in tumor cells may play a role in tumor development as a result of the instability of the tumor genome.

### Drug susceptibility analysis of ABI3BP

**Figure 11:**
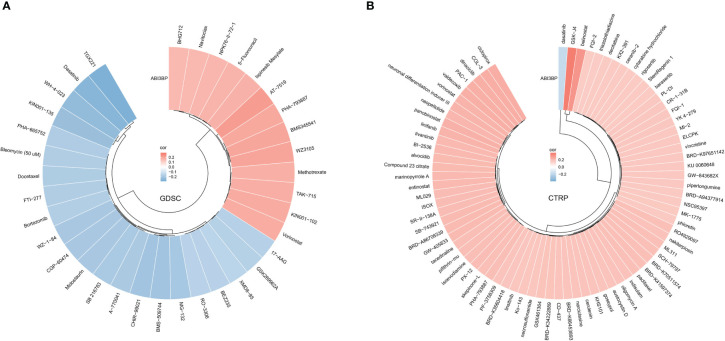
Relationship between ABI3BP expression and drug sensitivity. Correlation between ABI3BP expression and drug sensitivity to GDSC **(A)** and CTRP drugs **(B)** in pan-cancer.

We searched the GDSC website for the relationship between ABI3BP mRNA expression and drug susceptibility; our results indicated that ABI3BP was sensitive to AT-7519, PHA793887, and 5-Fluorouraci, but resistant to TGX221, WH-4-023, and Dasatinib (**Figure 11A**). In addition, consistent with GDSC results, the CTRP website indicated that ABI3BP was sensitive to GSK-J4 and Belinostat but resistant to Dasatinib (**Figure 11B**).

## Discussion

With the progression of time, an increasing number of exogenous and endogenous factors contribute to cancer development, making it imperative to develop more effective and safer treatments. Tumor immunotherapy is a form of cancer treatment that reactivates and maintains the tumor-immune cycle and restores the body’s natural anti-tumor immune response (24). Examples include inhibitors of immune checkpoints (25), therapeutic antibodies (26), cancer vaccines (27), and tiny-molecule chemical inhibitors (28). The unanticipated discovery of new effective prognostic indicators or treatment targets may contribute to decreasing cancer mortality and increasing cancer survivability.

Multiple organs, such as the lungs and heart, express ABI family member 3 binding protein (ABI3BP), an extracellular matrix protein (8). A study demonstrated that ABI3BP inhibits thyroid cancer cell viability, migration, invasion, and tumor growth. Similarly, we discovered that forced expression of ABI3BP in gallbladder cancer decreased tumor activity (29). However, the mechanism through which ABI3BP inhibits tumorigenesis remains unknown. Consequently, we analyzed the expression of mRNA and protein of ABI3BP in pan-carcinoma using public databases and immunohistochemistry. Normal tissues were shown to express ABI3BP much more than malignant tissues. In certain cancer patients, ABI3BP was identified as an independent prognostic factor, and its high expression was linked with a better prognosis. ABI3BP has superior diagnostic and prognostic performance for the prognosis of patients, and it is closely related to the clinical stage, grade, gender, and age of patients. A malignant tumor is a complicated illness marked by alterations in the expression and function of a variety of immune-related components (30). On the one hand, the immune system performs a significant anti-tumor function; It also contributes to the incidence and growth of tumors, making it a double-edged sword. ABI3BP was associated with an increase in B cell, T cell CD4+, T cell CD8+, Macrophage, and Neutrophil infiltration of tumor cells, as determined by additional analyses of immune infiltration. ABI3BP expression was also linked to immune checkpoint genes, TMB, MSI, and other tumor heterogeneity factors. Our research indicates that ABI3BP may be an essential immune target during tumor development. In order to comprehend the synergistic effect of ABI3BP on cancer, we screened 20 ABI3BP-related genes as a gene set for a comprehensive study. We discovered that the gene set was significantly associated with biological processes such as apoptosis, cell cycle, DNA damage, and EMT. In addition, copy number variations of gene sets were prevalent in pan-cancers. The close relationship between ABI3BP and patient prognosis suggests that ABI3BP is involved in regulating cancer-related functional status. Due to the heterogeneity of tumors, chemotherapy remains an essential treatment for metastatic or advanced lung cancer to prevent recurrence and prolong survival. AT7519 is a small-molecule cyclin-dependent kinase inhibitor. As demonstrated by scientific studies, multiple oncogenic signaling pathways have been identified as how AT7519 enhances cisplatin’s efficacy in treating ovarian cancer (31). AT7519 effectively overcomes chemotherapy resistance in the colon and cervical cancer by inhibiting cyclin-dependent kinases. ABI3BP is sensitive to AT7519, indicating that it may play a role in the cell cycle of tumor cells. In conclusion, the association between ABI3BP and patient prognosis, clinical data, gene alterations, and tumor immune infiltration provides novel information for future clinical diagnosis and therapy.

This work contributes to our knowledge of the relationship between ABI3BP and pan-cancer, although it has several drawbacks. We first investigated the expression of ABI3BP in pan-carcinoma and immune-related activities. However, further fundamental experimental study is required to comprehend the molecular mechanism of ABI3BP in the growth of tumors. Second, we have only investigated the abnormal expression of ABI3BP-related genes; the gene-protein signaling pathway changes resulting from ABI3BP-related gene changes have yet to be thoroughly investigated.

In conclusion, our findings demonstrate that ABI3BP expression levels vary widely among human cancers and correlate with clinical case characteristics and prognosis in pan-cancer patients. Immune infiltration of tumor cells is tightly associated with ABI3BP expression. Finally, we conducted a comprehensive immunological examination of ABI3BP, demonstrating its potential relevance as a prognostic biomarker for patients.

## Data availability statement

The raw data supporting the conclusions of this article will be made available by the authors, without undue reservation.

## Ethics statement

The studies involving human participants were reviewed and approved by the Ethics Committee of the Qingdao Municipal Hospital. The patients/participants provided their written informed consent to participate in this study.

## Author contributions

YF provided the article design and data acquisition. FT, HQ provided research materials and statistics. HT provided administrative support and article design. All the authors contributed to the manuscript writing and final review. All the authors agreement to be accountable for all aspects of the work in ensuring that questions related to the accuracy or integrity of any part of the work are appropriately investigated and resolved.

## Supplementary material

Supplementary Figure 1 Univariate Cox regression analysis of ABI3BP. **(A)**Forest plot shows the results of univariate cox regression of ABI3BP on DFI in TCGA pan-cancer. **(B, C)** K-M survival curves show the DFI prognosis survival of LIHC and ESCA patients in the ABI3BP high and low expression groups. **(D)**Forest plot shows the univariate cox regression results of ABI3BP on PFI in TCGA pan-cancer. The **(E-J)** K-M survival curve showed the PFI prognosis survival of ACC, KIRP, KIRC, LIHC, SARC, KICH patients in the ABI3BP high and low expression groups.

Supplementary Figure 2 Relationship between ABI3BP and tumor immune infiltration. Immune cell infiltration levels were analyzed by **(A)** MCPCOUNTER, **(B)** xCell, **(C)** EPIC, and **(D)** QuanTIseq algorithms.

Supplementary Table 1 Abbreviation.
