# Supplementary figures and images for: A pan-cancer analysis of ABI3BP: a potential biomarker for prognosis and immunoinfiltration
Source: Front Oncol. 2023 May 1;13:1159725. doi: 10.3389/fonc.2023.1159725 (PMC10183607; doi:10.3389/fonc.2023.1159725)

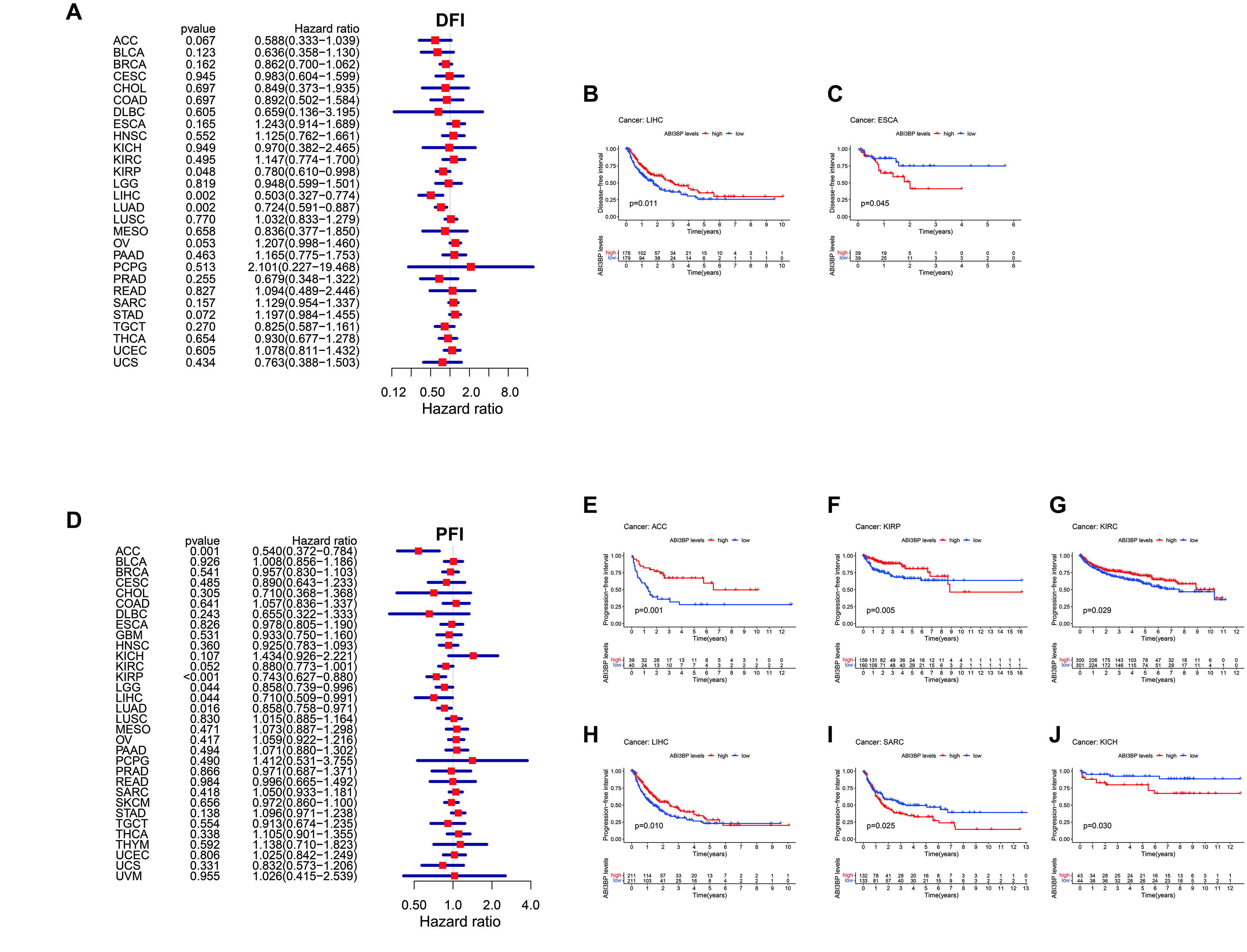

Supplement: Supplementary Figure 1 — Univariate Cox regression analysis of ABI3BP. (A)Forest plot shows the results of univariate cox regression of ABI3BP on DFI in TCGA pan-cancer. (B, C) K-M survival curves show the DFI prognosis survival of LIHC and ESCA patients in the ABI3BP high and low expression groups. (D)Forest plot shows the univariate cox regression results of ABI3BP on PFI in TCGA pan-cancer. The (E-J) K-M survival curve showed the PFI prognosis survival of ACC, KIRP, KIRC, LIHC, SARC, KICH patients in the ABI3BP high and low expression groups. [file Image_1.jpeg]

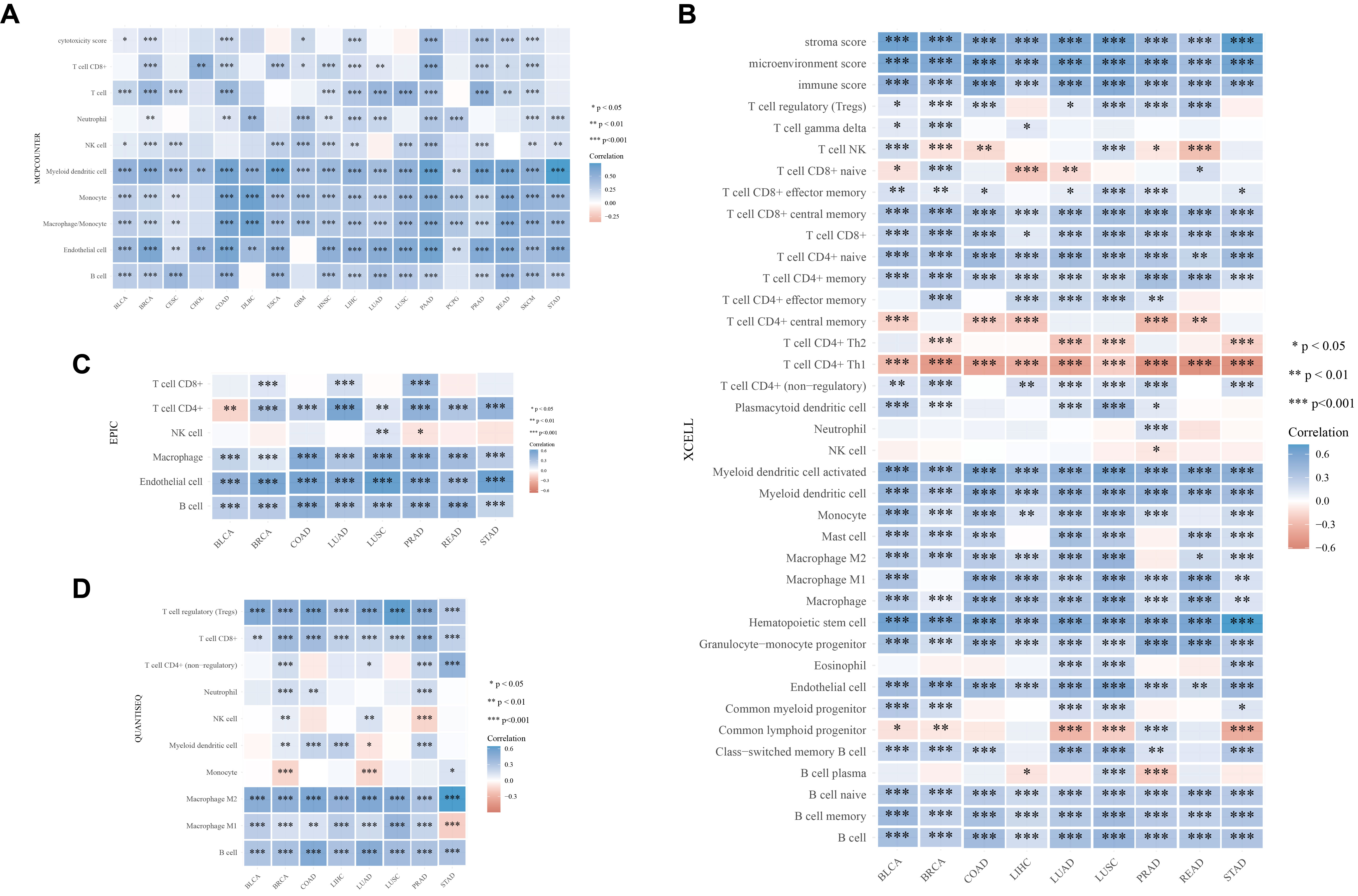

Supplement: Supplementary Figure 2 — Relationship between ABI3BP and tumor immune infiltration. Immune cell infiltration levels were analyzed by (A) MCPCOUNTER, (B) xCell, (C) EPIC, and (D) QuanTIseq algorithms. [file Image_2.jpeg]
