# Supplementary material for: A pan-cancer analysis of ABI3BP: a potential biomarker for prognosis and immunoinfiltration
Source: Front Oncol. 2023 May 1;13:1159725. doi: 10.3389/fonc.2023.1159725 (PMC10183607; doi:10.3389/fonc.2023.1159725)
Supplement: Supplementary Table 1 — Abbreviation. [file Table_1.docx]

| Supplementary Table. 1 Abbreviation | |
| --- | --- |
| Full Name | Abbreviation |
| area under the curve | AUC |
| receiver operating characteristic | ROC |
| tumor immune estimation resource | TIMER |
| copy number variation | CNV |
| gene set variation analysis | GSVA |
| gene set cancer analysis | GSCA |
| tumor Mutation Burden | TMB |
| microsatellite Instability | MSI |
| mismatch repair | MMR |
| follicular helper T cell | Tfh |
| Natural killer T cell | NKT |
| Regulatory T cells | Treg |
| Adrenocortical Cancer | ACC |
| Bladder Cancer | BLCA |
| Breast Cancer | BRCA |
| Cervical Cancer | CESC |
| Bile Duct Cancer | CHOL |
| Colon Cancer | COAD |
| Colon and Rectal Cancer | COADREAD |
| Large B-cell Lymphoma | DLBC |
| Esophageal Cancer | ESCA |
| Glioblastoma | GBM |
| lower grade glioma and glioblastoma | GBMLGG |
| Head and Neck Cancer | HNSC |
| Kidney Chromophobe | KICH |
| Kidney Clear Cell Carcinoma | KIRC |
| Kidney Papillary Cell Carcinoma | KIRP |
| Acute Myeloid Leukemia | LAML |
| Lower Grade Glioma | LGG |
| Liver Cancer | LIHC |
| Lung Adenocarcinoma | LUAD |
| Lung Cancer | LUNG |
| Lung Squamous Cell Carcinoma | LUSC |
| Mesothelioma | MESO |
| Ovarian Cancer | OV |
| Pancreatic Cancer | PAAD |
| Pheochromocytoma & Paraganglioma | PCPG |
| Prostate Cancer | PRAD |
| Rectal Cancer | READ |
| Sarcoma | SARC |
| Melanoma | SKCM |
| Stomach Cancer | STAD |
| Stomach and Esophageal carcinoma | STES |
| Testicular Cancer | TGCT |
| Thyroid Cancer | THCA |
| Thymoma | THYM |
| Endometrioid Cancer | UCEC |
| Uterine Carcinosarcoma | UCS |
| Ocular melanomas | UVM |
